# Women Living with HIV in Italian Prison Settings: Results from the Gender-Specific ROSE Network

**DOI:** 10.3390/v15020497

**Published:** 2023-02-10

**Authors:** Elena Rastrelli, Vito Fiore, Roberto Ranieri, Emanuele Pontali, Tullio Prestileo, Giorgio Barbarini, Anna Maria Ialungo, Serena Dell’Isola, Andrea De Vito, Matteo Bolcato, Giordano Madeddu, Giulio Di Mizio, Giulio Starnini, Sergio Babudieri

**Affiliations:** 1 Medicina Protetta-Unit of Infectious Diseases, Belcolle Hospital, 01100 Viterbo, Italy; 2 Infectious and Tropical Disease Clinic, Department of Medical, Surgical and Experimental Sciences, University of Sassari, 07100 Sassari, Italy; 3 Penitentiary Infectious Diseases Unit, A.O. Santi Paolo e Carlo, University of Milan, 20122 Milan, Italy; 4 Infectious Disease Unit, Galliera Hospital, 16128 Genoa, Italy; 5 ARNAS, Ospedale Civico-Benfratelli Palermo, 90127 Palermo, Italy; 6 Department of Infectious Disease, IRCCS San Matteo, 27100 Pavia, Italy; 7 Legal Medicine, University of Padua, 35121 Padova, Italy; 8 Forensic Medicine, Department of Law, Magna Graecia, University of Catanzaro, 88100 Catanzaro, Italy; 9 Scuola di Formazione Permanente in Medicina e Sanità Penitenziaria Simspe Onlus, 01100 Viterbo, Italy

**Keywords:** prison settings, health disparities, HIV, HBV, HCV

## Abstract

Background: Incarcerated women are a minority in the Italian prison population. The lack of prevention and awareness of HIV infection and the lack of access to treatment make the treatment path difficult. Methods: we conducted a multi-center study including incarcerated women living with HIV (WLWH). Results: The study included 85 WLWH with a mean age of 41.7 ± 8.7 years, and 58.8% (50/85) of them were Italian. Principally, HIV transmission was related to sexual intercourse, 47% of all patients were PWIDs, and 62.5% of them were on opioid substitution therapy (OST). Overall, 56.4% of the included patients had a CD4+ cell count of >500 cells/mmc. Among the participants, 92.9% were on antiretroviral therapy, 87.3% had treatment before incarceration, and 83.5% were virologically suppressed. Among the 13 non-virally-suppressed patients, 53.8% were unaware of their serological status before incarceration and had started HAART but were still not virologically suppressed; 46.2% (6/13) had a lack of compliance or had suspended the treatment before incarceration and restarted it after admission. All patients with chronic hepatitis C underwent treatment with direct-acting antivirals and reached a sustained virological response. Conclusions: the detention of these women could represent an occasion for the patients’ healthcare provision and use, and the creation of a gender-specific network can be an effective strategy for reaching this population.

## 1. Introduction

Worldwide, women are a minority in prison populations [1,2]. This may suggest the reason for the insufficient organization of health screening services and the removal of these women from their city of origin and, therefore, from their family (particularly from their children). Their shorter detention time due to minor offenses is an obstacle to the proper taking charge of their care in prison and for therapeutic continuity planning after their discharge [1,2].

Incarcerated women are more likely to develop blood-borne virus (BBV) infections.

As per previous Italian Ministry of Health data, the HIV prevalence among incarcerated women as well as the risk of other BBV infections were higher than those among incarcerated men and the general population [3].

Furthermore, incarcerated women represent <5% of prison population [4,5,6], making them even harder to reach. In fact, all of the most recent national literature highlighted the lack of data regarding BBV among incarcerated women [4,5,6].

The frequent association of these diseases with drug addiction, prostitution, histories of violence, and social fragility makes incarcerated women a target population for healthcare provision, screening, and highly active antiretroviral therapy (HAART) initiation and adherence support [7].

There are no official national data regarding incarcerated women living with HIV (WLWH). For this reason, the Italian Society of Penitentiary Healthcare (SIMSPe) created a gender-specific network (ROSE network) to maximally increase healthcare provision and use among incarcerated women.

The aim of our study is to report the ROSE network’s preliminary data regarding incarcerated WLWH: risk factors, HAART coverture and adherence, as well as other BBVs co-infections among the female prison population.

## 2. Patients and Methods

### 2.1. Study

The ROSE HIV network was set up involving 21 Italian penitentiary institutes located in 12 Italian regions. This is a gender-specific network focused on incarcerated women that aims to better address the issue of infectious diseases in this population, particularly blood-borne viruses (BBV), such as HIV, HBV, and HCV.

The inclusion criteria for this study were as follows: WLWH, adult (>18 years old), and incarcerated at the time of the study. Information on the patients’ medical history, demographics, virological data, the presence of other BBV (HCV, HBV), previous serological awareness before incarceration, and whether they were people who injected drugs (PWIDs) was obtained from the patients’ medical records based on the Addiction Services evaluation, which is available in every Italian detention center. Addiction services apply to medical groups that are involved in dependency evaluation (e.g., the use of illicit drugs or alcohol abuse) during prison admission and in treatment and rehabilitation provision during their prison stay.

### 2.2. Statistical Analysis

The data distribution was evaluated with the Kolgomorov–Smirnov test before elaboration. Demographic variables were summarized as absolute and relative (percentage) frequencies, whereas quantitative variables were described as means (standard deviations, SD) or medians (interquartile ranges, IQR) according to whether the distributions were parametric or non-parametric. Data were elaborated as numbers in total (percentages), means ± standard deviations, and medians (IQR). Categorical variables were compared with a chi-squared or Fischer exact test when appropriate.

A two-tailed *p*-value less than 0.05 was considered statistically significant. All statistical computations were carried out with the statistical software STATA version 16 (StatsCorp, College Station, TX, USA).

### 2.3. Ethical Issues

This study was conducted in accordance with the Declaration of Helsinki. All studies regarding the Italian Penitentiary System have been approved by the Ethics Committee of the University of Rome “Tor Vergata” [Registro sperimentazioni 73/05].

## 3. Results

### 3.1. Patients’ Demographics and Clinical Features

Overall, 85 WLWH were included whose mean age was 41.7 + 8.7 years and of whom 58.8% (50/85) were Italian. Regarding HIV transmission, 47% (40/85) were PWIDs, and 62.5% (25/40) of them were on OST. Almost all other cases were sexually transmitted. Most patients (48; 56.4%) had a CD4+ cell count above 500 cells/mmc. The demographics, clinical features, and the current highly active antiretroviral therapy (HAART) of patients included in our study have been reported in Table 1.

When evaluating the relationship between nationality and transmission patterns, drug injection was a more frequent risk factor among Italian patients than among those of other nationalities [Italian nationality vs. non-Italian nationality = 32 (80%) vs. 8 (20%), *p* = 0.0002].

### 3.2. Treatment Coverture, Viral Suppression, Genotypes, and Level of Awareness

Overall, 92.9% (79/85) of patients were on HAART. Among them, 87.3% (69/79) had treatment before incarceration and 83.5% (66/79) were virologically suppressed at the time of our evaluation. Regarding the non-virally-suppressed patients on HAART (13/79; 16.4%), 53.8% (7/13) were unaware of their serological status before incarceration and had started HAART but were still not virologically suppressed, while 46.2% (6/13) had a lack of compliance or had suspended the treatment before incarceration and restarted it after admission. When comparing the differences in HAART compliance between PWIDs vs. non-PWIDs, they were 5 (11.3%) vs. 3 (7.7%), *p*-value = 0.71. Furthermore, there was no significant difference in awareness among people diagnosed at the time of incarceration [PWIDs vs. non-PWIDs = 2 (4.5%) vs. 5 (12.2%), *p* = 0.25].

The HIV genotype was available only in 47% (40/85) of patients. Among them, mutations were present in 10% of cases: in 75% (3/4) of patients, there was NNRTI mutation (K103N), and one (25%) had both NRTI and INSTI mutation (A62AV, K65R, M184V, T66I, S147G).

### 3.3. Other Blood-Borne Virus Co-Infections and Treatments

Overall, 22.3% (19/85) of patients had HBV co-infection. Among them, 15.8% (3/19) had chronic hepatitis B (CHB) and 84.2% (16/19) had occult B infection (OBI). All patients with CHB were in HAART and had negative HBV-DNA.

HCV seroprevalence was in 44.7% (38/85) of patients, 78.9% (30/38) of which were PWIDs, and 52.6% (20/38) had positive HCV-RNA. Of the latter, 20% (4/20) were new diagnoses. The most frequent genotype was 1a (13/20; 65%). All new diagnoses had positive HCV-RNA. Only one patient had liver cirrhosis. Although the HCV seroprevalence was significantly higher among PWIDs [PWIDs vs. non-PWIDs = 30 (75%) vs. 8 (17.7%), *p* < 0.00001], there was no statistically significant difference both in active infections [PWIDs vs. non-PWIDs = 15 (50%) vs. 3 (37.5%), *p* = 0.69] and levels of awareness [PWIDs vs. non-PWIDs = 3 (2%) vs. 1 (2%), *p* = 1.0] between the two groups. Data on patients with viral hepatitis co-infections have been reported in Table 2.

Out of the 18 patients who tested negative for HCV-RNA, 66.6% (12/18) had been previously treated with second-generation direct-acting antivirals (DAAs) and only one was a spontaneous eradication.

All HCV-RNA positive patients underwent DAAs treatment and achieved SVR12.

**Figure 1:**
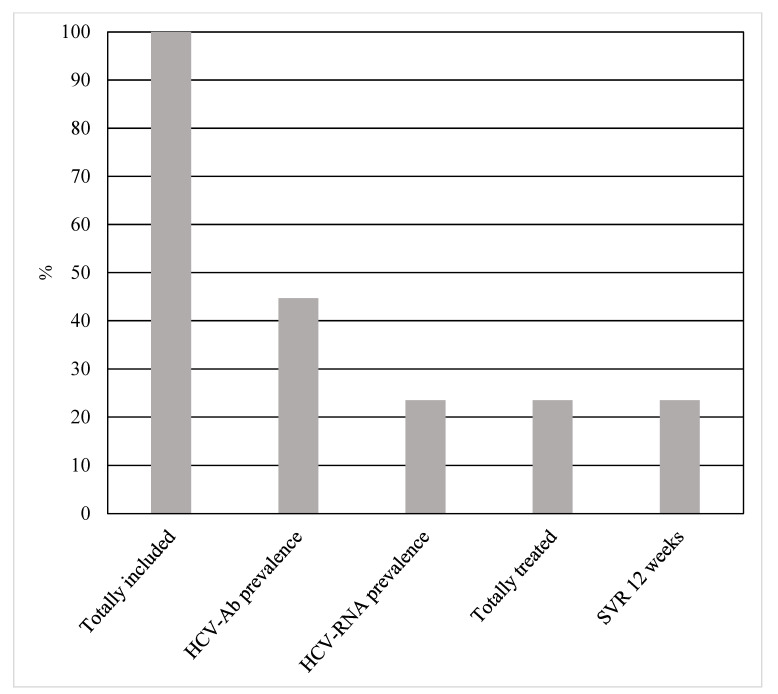
HCV cascade of care among 85 incarcerated women living with HIV.

The HCV cascade of care among WLWH included in our study has been reported in Figure 1.

## 4. Discussion

All of the national literature on prison settings highlights the low percentage of women inclusion in studies (4–6). To our knowledge, this is the first and the biggest Italian study including only incarcerated WLWH. Women represent <5% of the incarcerated population [4,6,8] and HIV national prevalence in these settings has been reported as 3.8% (overall, including men and women). It follows that incarcerated WLWH represent an even harder-to-reach sub-population. This is the reason why specific networks should be created to obtain maximal healthcare provision and use among incarcerated women.

When evaluating risk factors, most of the included women were infected through sexual transmission and <50% were PWIDs. This could be considered in line with the available national literature, reflecting the high variable range of PWIDs described by previous studies (PWIDs = 11.8–91.1%) [9,10,11,12,13,14].

Of the included patients, 36.8% were unaware of their serological status prior to their incarceration and had started treatment during detention. Furthermore, WLWH were reported to have suspended HAART before incarceration and to have restarted HAART after prison admission. This confirms that prison settings represent an extraordinary occasion for healthcare provision, guaranteeing the elimination of health disparities in underserved populations [15,16,17,18]. Furthermore, this highlights the importance of the linkage to care in territorial services, reducing losses in follow-up and reducing HAART discontinuations.

Regarding other BBVs, HCV was the most common co-infection (~45%). This slightly differs from the available literature, where the HCV positivity described among incarcerated people living with HIV (PLWH) ranges from 54.1 to 90.8% [9,10,11,12,13,14]. Furthermore, this was more than twice higher than the general female incarcerated population [19]. Instead, the level of awareness of HCV infection was not different between PWIDs and non-PWIDs (*p* = 1.0). This datum differs from our previous data on the general incarcerated population, where the level of awareness was statistically significant (*p*-value < 0.001) (5). Probably, this is related to the fact that only PLWH were included in the present study and that the HCV test was performed earlier than it is in the general prison population.

Surprisingly, >50% of the included women tested positive for HCV-RNA. This confirms the necessity of tailored interventions for the female prison population. All treated patients reached SVR12, confirming both the feasibility and efficacy of DAAs treatments previously reported by the literature in field [4,6,19,20].

Overall, the rate of HBV positivity was ~22%. Our data could be considered concordant with the available literature, which describes a wide range of HBV positivity among PLWH (2.4–81.2%).

## 5. Conclusions

Incarcerated WLWH are a fragile sub-population with particular needs, given their presence in small groups throughout the national prison system. Detention could represent an occasion for healthcare provision and use, and the creation of a gender specific network can be an effective strategy for reaching this population.

## 6. Limitations of the Study

Some limitations should be acknowledged regarding our study. First, most of the data were obtained from patients’ medical records (retrospective analysis). Second, our data came only from self-included centers. According to the Italian Society of Penitentiary Medicine, the HIV antibody seroprevalence in Italian prison settings is 1.3%. However, punctual data on the female prison population are not reported [21,22]. Only WLWH were included in our work. For this reason, data from more penitentiary centers would be needed to better define healthcare provision and use in the whole female prison population.

Unfortunately, we do not have data regarding the infection timeline because this is not a part of the standard anamnesis during prison admission. The CD4+ count was related to the available measures after admission.

Data on infection pathways and PWID definition came from patients’ medical records. For this reason, there could have been biases in identifying the patients’ risk factors.

## Figures and Tables

**Table 1:** Characteristics of the 85 incarcerated women living with HIV included in our study.

Variable	Result
Nationality	
Italy, n (%)	50 (58.8)
Africa, n (%)	25 (29.4)
South America	4 (4.7)
Slovakia	2 (2.3)
Romania	2 (2.3)
Czech Republic	1 (1.2)
Albania	1 (1.2)
Age, mean ± SD	41.7 ± 8.7
Risk factor	
Drug injection, n (%)	40 (47)
Sexual transmission, n (%)	44 (51.8)
Blood transfusion	1 (1.2)
CD4+ count, median (IQR)	573 (362–696)
On HAART, n (%)	79 (92.9)
INSTI-based, n (%)	28 (32.9)
PI-based, n (%)	42 (49.4)
NNRTI-based, n (%)	7 (8.2)

SD: standard deviation; IQR: interquartile range; HAART: highly active antiretroviral therapy; INSTI: integrase strand transfer inhibitor; PI: protease inhibitor; NNRTI: non-nucleoside reverse transcriptase inhibitor.

**Table 2:** Viral hepatitis co-infections in 85 incarcerated women living with HIV.

Variable	Result
HCV-Ab positive, n (%) Active infection, n (%)	38 (44.7) 20 (52.2)
HBV positivity, n (%) HBsAg positive, n (%) OBI, n (%)	19 (22.3) 3 (15.8) 16 (84.2)

HCV-Ab: hepatitis C virus antibody; HBV: hepatitis B virus. HBsAg: hepatitis B surface antigen; OBI: occult B infection.

## Data Availability

All data are available within the manuscript.
